# Viral Hepatitis, Cholesterol Metabolism, and Cholesterol-Lowering Natural Compounds

**DOI:** 10.3390/ijms23073897

**Published:** 2022-03-31

**Authors:** Je-Wen Liou, Hemalatha Mani, Jui-Hung Yen

**Affiliations:** 1 Department of Biochemistry, School of Medicine, Tzu Chi University, Hualien 97004, Taiwan; jwliou@mail.tcu.edu.tw; 2 Institute of Medical Sciences, Tzu Chi University, Hualien 97004, Taiwan; 104325118@gms.tcu.edu.tw; 3 Department of Molecular Biology and Human Genetics, Tzu Chi University, Hualien 97004, Taiwan

**Keywords:** hepatitis virus, cholesterol metabolism, HMG-CoA reductase, phytochemicals

## Abstract

Hepatitis is defined as inflammation of the liver; it can be acute or chronic. In chronic cases, the prolonged inflammation gradually damages the liver, resulting in liver fibrosis, cirrhosis, and sometimes liver failure or cancer. Hepatitis is often caused by viral infections. The most common causes of viral hepatitis are the five hepatitis viruses—hepatitis A virus (HAV), hepatitis B virus (HBV), hepatitis C virus (HCV), hepatitis D virus (HDV), and hepatitis E virus (HEV). While HAV and HEV rarely (or do not) cause chronic hepatitis, a considerable proportion of acute hepatitis cases caused by HBV (sometimes co-infected with HDV) and HCV infections become chronic. Thus, many medical researchers have focused on the treatment of HBV and HCV. It has been documented that host lipid metabolism, particularly cholesterol metabolism, is required for the hepatitis viral infection and life cycle. Thus, manipulating host cholesterol metabolism-related genes and proteins is a strategy used in fighting the viral infections. Efforts have been made to evaluate the efficacy of cholesterol-lowering drugs, particularly 3-hydroxy-3-methylglutaryl coenzyme A (HMG-CoA) reductase inhibitors, in the treatment of hepatitis viral infections; promising results have been obtained. This review provides information on the relationships between hepatitis viruses and host cholesterol metabolism/homeostasis, as well as the discovery/development of cholesterol-lowering natural phytochemicals that could potentially be applied in the treatment of viral hepatitis.

## 1. Introduction

Viral hepatitis is an inflammatory condition of the liver, caused by a viral infection. Although other viruses can sometimes cause liver inflammation, viral hepatitis is commonly caused by infections of hepatitis viruses. Five hepatitis viruses from distinct genetic backgrounds have been identified. Among these five, hepatitis A virus (HAV), a *Picornaviridae* virus; and hepatitis E virus (HEV), a member of *Hepeviridae*, can be transmitted via the fecal–oral route; these viruses are endemic in developing (low-income) countries [1]. They normally cause self-limiting hepatitis, and do not lead to fulminant liver failure. Chronic infections of these viruses only occur in rare cases in immune-compromised patients. On the other hand, the hepatitis B virus (HBV), a double-stranded DNA virus belonging to *Hepadnaviridae*; and the hepatitis C virus (HCV), a positive-sense single-stranded RNA virus belonging to *Flaviviridae*, cause acute and chronic hepatitis. Chronic viral hepatitis progressively results in liver fibrosis, cirrhosis, and, in many cases, hepatocellular carcinoma (HCC) [2]. The hepatitis D virus (hepatitis delta virus, HDV) is a negative-sense single-stranded RNA virus belonging to the genus *Deltavirus*. It is a satellite virus that has no independent life cycle and can only propagate depending on the function of HBV for its replication and expression. This means that HDV can only replicate in the host with a persistent HBV infection. The co-infection of HBV and HDV causes the most severe form of viral hepatitis [3]. Lipid metabolism in cells is highly related to inflammation and the development of cancers; host lipid metabolism has been linked to viral infection, replication, and assembly [4,5]. For hepatitis viruses that normally cause acute hepatitis only, altered lipid profiles have been observed in children with acute hepatitis induced by HAV infections [6]; decreased cholesterol levels and serum lipid concentrations were measured in HEV-infected cells, and in patients, respectively [7]. For viruses that cause chronic hepatitis, research shows that host lipid metabolism reprogramming is associated with HBV and HCV infections in the progression to severe liver disease [8,9]. Lipid droplets (LDs, also referred to as lipid bodies) are organelles that play important roles in lipid metabolism, energy homeostasis, and intracellular transport. They also have multiple roles in infections and inflammation. The LDs involve the storage of lipids and cholesterol in cells, and are associated with a variety of viruses, including *Flaviviridae* members dengue virus [10], rotavirus [11,12], reovirus [13], and Zika virus [4] for viral particle formations. It has been indicated that the replication and inflammatory mediator productions of other viruses, such as SARS-CoV-2, are fueled by LDs [14]. The interactions between HCV proteins and host cellular LDs have been determined as necessary for infectious particle productions [15,16,17] and the level of cellular cholesterol is important in viral life cycles [18]. In this review, the links between cholesterol metabolism and viral hepatitis are discussed. As research shows that a range of phytochemicals are effective at reducing cholesterol levels in the body and in the cells, it is possible to apply natural phytochemicals to manipulate cholesterol metabolism as a treatment strategy for hepatitis viral infections. The phytochemicals with cholesterol-lowering activities, as well as their identified antiviral effects, are also summarized in this review.

## 2. Cholesterol Homeostasis

Cholesterol is one of the major components of mammalian biological membranes. It is required for proper membrane functioning, such as permeability, fluidity, and organelle identity. It is also a crucial biomaterial in maintaining structures and controlling activities of membrane proteins. In addition, cholesterol is a precursor for almost all steroid hormones [19]. Cells maintain their cholesterol homeostasis through complicated transcriptional and posttranscriptional mechanisms [20]. It has been suggested that mammalian cells are able to synthesize cholesterol [21], even though a large quantity of cholesterol can be absorbed by the gastrointestinal tract [22]. On the other hand, only hepatocytes are capable of degrading and eliminating cholesterol in great quantities [22].

**Figure 1:**
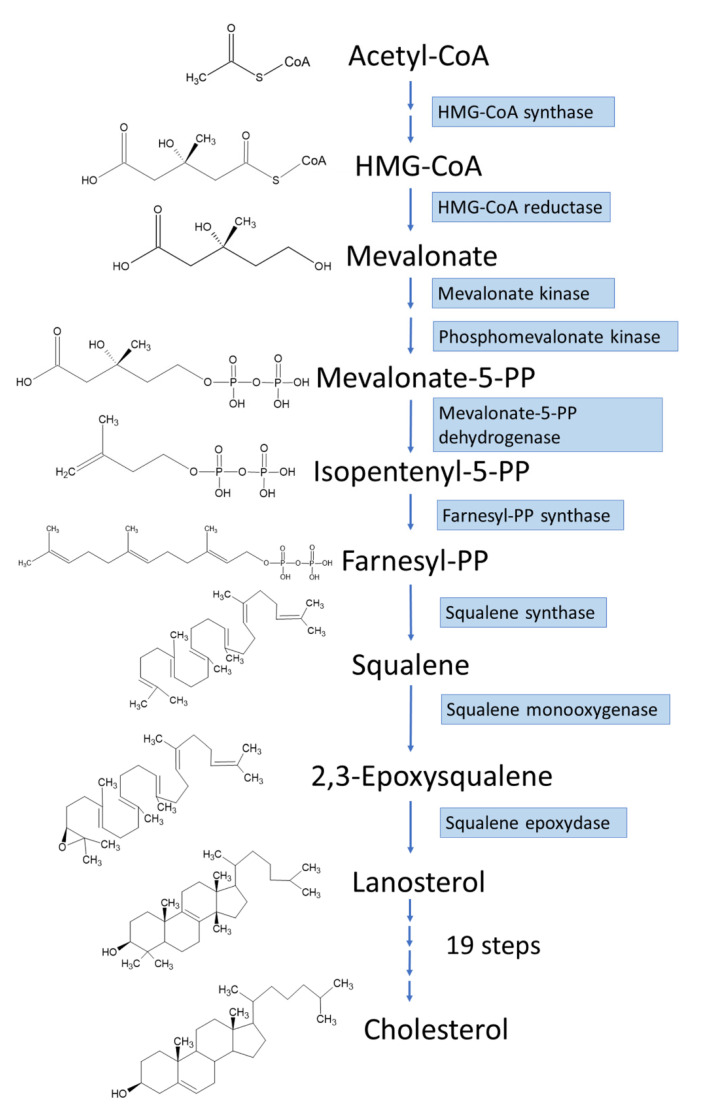
Cellular biosynthesis of cholesterol.

De novo biosynthesis of cholesterol in hepatic cells is a multistep biochemical process, starting from acetyl-CoA (Figure 1). The acetyl-CoA molecules are used as the substrate to synthesize 3-hydroxy-3-methylglutaryl coenzyme A (HMG-CoA) and the process is followed by the rate-limiting reaction in cholesterol biosynthesis catalyzed by HMG-CoA reductase to produce mevalonate, which is then processed into isopentenyl pyrophosphate. After that, the isopentenyl pyrophosphate is converted into lanosterol [23], which is further processed into cholesterol via a 19-step process [24]. Apart from de novo cholesterol biosynthesis, most cells acquire cholesterol from low-density lipoprotein (LDL) taken from the circulation via receptor-mediated endocytosis [25]. As cholesterol is an important component in the mammalian body, its homeostasis is crucial for proper cellular and systemic biological functions. Disturbance in cholesterol balance is suggested to be a significant factor, not only for cardiovascular disease [26,27], but also for a variety of other diseases, such as neurodegenerative diseases [28,29] and cancers [3,21,24]. In addition, cholesterol metabolism has great impact on the immune system and influence on antitumor immune responses [30].

Lipid rafts are cholesterol- and sphingolipid-enriched membrane domains containing specific proteins for specialized cellular functions. Lipid rafts are involved in the signal transduction in cancer cell survival, cell death, and metastasis [31]. Alterations of lipid rafts are also linked to age-associated neurodegenerative diseases, such as Alzheimer’s disease and Parkinson’s disease [32]. In addition, research shows that lipid raft signaling is involved in the pathogenesis of a variety of conditions, such as cardiovascular diseases, prion diseases, systemic lupus erythematosus, and HIV infection [33].

LDs are reservoirs for cholesterol and lipids in mammalian cells. They are already linked to inflammatory responses through the synthesis and metabolism of eicosanoids and to metabolic disorders, such as obesity, cancer, and atherosclerosis [34,35]. The relationships between infectious diseases and LDs have been documented. Viruses, bacteria, and parasites obtain substrates (directly or indirectly) from LDs, for energy metabolism, replication compartments, infectious particle assembly, membrane building blocks, and tools for host colonization and/or evasion of anti-inflammatory responses [36]. As mentioned previously, cholesterol and its metabolism are crucial for the life cycles of a variety of viruses and are linked to host inflammation; in this article, we discuss the roles played by cholesterol metabolism in viral hepatitis.

## 3. Hepatitis Viruses and Cholesterol Metabolism

Viral hepatitis is a condition of inflammation in the liver induced by viral infections. The most common viruses causing viral hepatitis are hepatitis A, B, C, D, and E viruses (characteristics of hepatitis viruses are summarized in Table 1), while other types of viruses, such as cytomegalovirus [37], Epstein–Barr virus [38], yellow fever virus [39], and herpes simplex virus [40], can also cause liver inflammation. HAV and HEV are the leading causes of acute viral hepatitis worldwide. They are present in the feces of infected individuals and are typically transmitted by ingestion of contaminated food or water. In most cases, the symptoms caused by infections with HAV and HEV are relatively mild, with most infected patients making a full recovery. People in low-income countries, where sanitary and conditions are poor, are easily infected with these two viruses [41]. On the other hand, HBV and HCV can cause both acute and chronic hepatitis. HBV, HCV, and HDV are usually transmitted through parenteral contact with infected body fluids, including receiving contaminated blood or blood products, and invasive medical procedures using contaminated equipment. For HBV, transmissions from mother to baby at birth and through sexual contact are also frequently reported. On the contrary, the sexual transmission of HCV is much less common. HDV is identified as a satellite virus with infections only occurring in patients already infected with HBV. The dual infection of HDV and HBV very often results in a more serious illness and worse outcome. As HDV relies on HBV for its propagation and life cycle, HBV vaccines are able to offer protection from HDV infections. 

### 3.1. Hepatitis A Virus and Hepatitis E Virus

HAV is a member of the *Hepatovirus* genus of *Picornaviridae* family. It is the only species known to infect humans in this genus [42,43]. HEV is classified as a member of the genus *Orthohepevirus*, in the family *Hepeviridae*. There are four species (A to D) in *Orthohepevirus*, yet only *Orthohepevirus A* infects humans [42]. HAV and HEV, despite significant differences in their evolutionary origins and genomic structures, tend to employ similar pathogenic strategies, and the diseases caused by these two viruses are clinically indistinguishable from each other. Both HAV and HEV are considered non-enveloped icosahedral viruses. As the lipid bilayer envelopes of viruses are easily disrupted in the environment, the lack of a lipid envelope offers both the viruses a more stable ability to spread in the environment. As a result, these two viruses frequently cause foodborne and waterborne outbreaks in many countries [42,44]. However, HAV and HEV isolated from the serum of patients suffering acute infections are “wrapped” with a hijacked layer of the host cell’s membrane, allowing the circulating virions to avoid host immune responses and attacks from neutralizing antibodies [45]. Hepatocytes are believed to be the principal sites of replication of HAV and HEV, and the virus particles are secreted to the intestinal tract via the biliary duct [46,47]. Experiments using cell culture and HAV-infected mice show that HAV is released across both the basolateral and apical membranes of polarized epithelial cells as enveloped HAV virions, and high concentrations of human bile acids (derivatives of cholesterol) convert quasi-enveloped HAV virions into naked, nonenveloped particles [47], as those found in the gut and stool of HAV infected patients. 

The relationships between HAV/HEV and cholesterol metabolism have been investigated. Altering lipid metabolism can be an efficient mean applied by the host to drive antiviral responses. Upregulation of intracellular LDs was identified as a weapon of the host cells to fight against viral infections [48]. Following infections of cells with either RNA viruses (e.g., Zika, dengue, influenza A) or DNA viruses (e.g., herpes simplex virus-1), LDs were rapidly upregulated; this response was also induced following stimulations with viral mimic agonists [48]. Indeed, it was also observed in the serum of young patients with self-limited acute viral hepatitis A; triglyceride, cholesterol, LDL, and Apo B levels elevated, while Apo A-I levels decreased [6]. It was found that HEV infection reduced cholesterol levels in cells, and decreased serum-lipid concentrations in patients [7]. Thus, cholesterol metabolism can be possibly applied as a target in antiviral strategies against HEV. It has been indicated that simvastatin treatment, which reduces intracellular cholesterol, increases viral release in vitro [7]. On the other hand, elevation of intracellular cholesterol with LDL, 25-hydroxycholesterol, or a cholesterol-elevating drug significantly reduces viral release due to enhanced lysosomal degradation of HEV [7].

### 3.2. Hepatitis B Virus

HBV can cause both acute and chronic liver inflammations. HBV infection is one of the major causes of HCC. Approximately 350 million people are chronically infected with HBV worldwide, and chronic HBV infections account for at least 50% cases of HCC globally [49]. Accumulated research results suggest that HBV infections affect lipid metabolism and could promote fatty acid synthesis [50,51]. Phosphatidylcholine is a major component of mammalian membrane and is a precursor for the synthesis of a number of signaling molecules [52]. The enhancement of phosphatidylcholine biosynthesis in hepatocytes by HBV infection was reported [53]. Cholesterol and LDs are also heavily impacted/controlled by HBV infection. Research shows that HBV infection in hepatocyte cultures is dependent on the presence of cholesterol in the viral envelope. According to a previous study, the depletion of cholesterol from HBV purified from the plasma of infected patients led to a significantly reduced ability of infection, but the binding of the virus to the cells was not affected [54]. These data indicate that the cholesterol content in the viral envelope is important for later steps in viral uptake. An animal model study using HBV-infected humanized mice revealed a significant increase in human genes related to the uptake, biosynthesis, and transcriptional regulation of cholesterol. These genes include those expressing LDL receptor (LDLR), HMG-CoA reductase, and sterol regulatory element binding protein-2 (SREBP-2) [55]. The effects of HBV infection on host cellular lipid metabolism have also been investigated. Research shows that altered lipid metabolisms in host cells are associated with HBV proteins. Several reports have suggested the roles played by the HBV X protein regarding this issue. Kim et al. demonstrated lipid accumulation and expression of lipid metabolic genes in HBV X protein-expressing cells and transgenic mice [56]. The results indicated that overexpression of HBV X protein induced hepatic lipid accumulation and this effect was linked to transcriptional activation of SREBP-1 and PPAR-γ [56]. Liver fatty acid binding proteins (FABPs) are a family of cytoplasmic proteins involved in lipid uptake, transport, and metabolism [57]. Wolfrum et al. reported that overexpression of FABP1 significantly increased the rate of fatty acid uptake [58]. An investigation by Wu et al. found that the expression level of FABP1 was elevated in the HBV-producing hepatoma cell line, and this FABP1 regulation was mediated by the HBV X protein [59]. 

In 2013, Li et al. used adenovirus to send the HBV genome into hepatocyte HepG2 and observed an increased hepatic cholesterol accumulation. In addition, the infection of HBV-containing adenovirus was able to enhance the mRNA and protein levels of LDLR and HMG-CoA reductase in cells. These inductive effects were possibly due to suppression of TLR2 expression levels by siRNA [60]. Selitsky et al. applied a mixture of genomic, molecular, and biochemical approaches to identify key miRNAs that drive lipid phenotypes of chronic viral hepatitis and hepatitis-associated HCC. Several miRNAs were identified as the candidate master regulators of pathways mediating cholesterol homeostasis in chronic hepatitis induced by HBV or HCV, and virus-infection associated liver cancer [2]. Experiments in Huh-7 hepatocytes found that miR-21, miR-27, and miR-224 suppressed cholesterol synthesis, and predictions showed that all three of these miRNAs target the 3′-untranslated region (3′-UTR) of HMG-CoA reductase [2]. 

Cell death-inducing DNA fragmentation factor-α (DFFA)-like effector (CIDE) proteins have been identified as lipid droplet-associated proteins that regulate lipid metabolism [61]. Yasumoto et al. reported that CIDEB and CIDEC positively regulate cellular LD size. Overexpression of CIDEB or CIDEC enhanced HBV production in HBV-infected cells (HepG2.2.15), and deficiency in CIDEB or CIDEC impaired HBV production and reduced the size of single LDs [62]. Yasumoto et al. also suggested that impairment of CIDE expression might stabilize an appropriate production level of HBV and inhibit excessive HBV productions [62]. Experimental results from HBV-inducible Hep38.7-Tet cells incubated with HBV suggested that HBV production may affect the expression of CIDE proteins in hepatocytes, and CIDE proteins were found to significantly enhance the activity of HBV core promoter [62]. CIDEs and HBV proteins might directly or indirectly interact with each other, and the balance between CIDE proteins and HBV production may contribute to moderate reduction in LD size in HBV-producing cells [62]. Downregulation of CIDE proteins might be a mean adopted by HBV to optimize the virus production levels to avoid tissue/cell damages and host immune clearance for persistent infection [62]. However, the clinical links between chronic HBV infection and the lipid accumulation in liver has not yet been fully understood. In population studies, it was found that HBV infection is associated with a low prevalence of fatty liver, hypertriglyceridemia, and metabolic syndrome [63], and positive results in serum HBV surface antigen are associated with low prevalence of metabolic syndrome [64]. Bile acid is mainly synthesized from cholesterol in the liver and plays a crucial role in lipid digestion and absorption. Human sodium taurocholate co-transporting polypeptide (NTCP, also known as solute carrier family 10 member 1, SLC10A1) mediates the entry of bile salts from hepatic portal blood into hepatocytes [65]. NTCP has also been identified as the receptor mediating species-specific entry of HBV into hepatocytes [66]. Thus, the association between bile acid and HCV infection was suggested. Evidence showed that the preS1 lipopeptide of HBV envelope protein efficiently blocked the uptake of bile salts by NTCP [67], suggesting a role played by HBV infection in limiting NTCP functions. The blocking of bile acid uptake promotes compensatory upregulation of cholesterol 7α-hydroxylase (CYP7A1), the rate-limiting enzyme in bile acid synthesis and HMG-CoA reductase thus increase the synthesis of bile acid and cholesterol in cells [68].

### 3.3. Hepatitis C Virus

The interplays between host lipid metabolism and HCV infection are the most studied among the hepatitis viruses. HCV infection alters the lipid and cholesterol metabolism in cells. Experiments using HepG2 cells transfected with the HCV core protein revealed that the expression of several genes related to lipid metabolism, including peroxisome proliferator-activated receptor (PPAR) α, multidrug resistance protein (MDR) 3, and microsomal triglyceride transfer protein (MTP), acyl-CoA oxidase (ACO) 1, and carnitine palmitoyl transferase-1 (CPT-1), were modulated by the expression of the HCV core protein [69]. It was also found that transient expression of the HCV core protein in mice downregulated the expression of lipid metabolism-associated genes, MDR2, CPT, AOX, and PPARα, leading to triglyceride (TG) accumulation and induction of oxidative stress [69]. Significant increases in serum levels of alanine aminotransferase, free fatty acids, TG, as well as the accumulation of hepatic lipid droplets, were observed in mice infected with HCV NS5A-expressing lentivirus [70]. In vitro and in vivo experiments showed that HCV NS5A was able to inhibit phosphorylation of AMP-activated protein kinase (AMPK), a key player in controlling cellular energy homeostasis by activating glucose and fatty acid uptake and oxidation [71]. HCV NS5A can also increase the expression levels of SREBP-1c, acetyl-coenzyme A carboxylase 1 (ACC1) and fatty acid synthase (FASN) [70].

**Figure 2:**
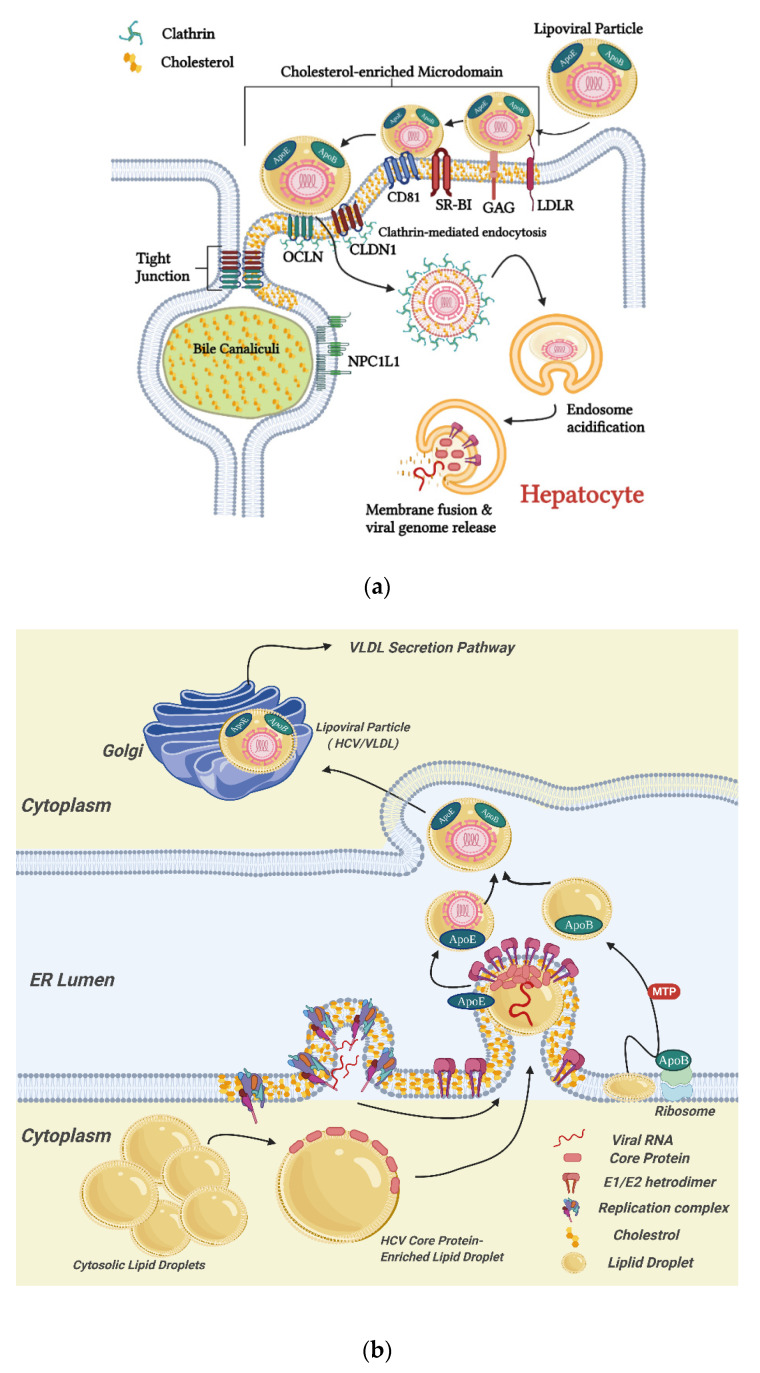
Schematic diagrams of the HCV entry into hepatocyte (**a**) and the replication, assembly, and secretion of HCV (**b**). (**a**) HCV entry into hepatocytes is a sequential process in which multiple factors, including glycosaminoglycan (GAG), low-density lipoprotein receptor (LDLR), scavenger receptor class B type I (SR-BI), CD81, tight junction proteins, claudin-1 (CLDN1), and occludin (OCLN), as well as Niemann–Pick C1-like 1 (NPC1L1), are involved [73,87,88]. The process starts with the binding of the lipoviral particle (LVP) to GAG and LDLR, followed by the interactions with SR-BI, CD81, CLDN1, and OCLN, leading to viral internalization in cholesterol-enriched microdomains (lipid rafts) of the membranes via a clathrin-mediated endocytosis; (**b**) HCV viral proteins induce rearrangement of ER membranes and form replication complexes [15]. Localization of the HCV polyprotein to cholesterol-enriched membrane fractions is required for the polyprotein cleavage [89], and the replication complexes are associating with cholesterol-enriched domains [83]. New viral genomic RNA is synthesized by the replication complexes assembled by non-structural (NS) proteins of HCV, and the newly produced HCV core protein is translocated to LDs. Virion assembly occurs on core protein-enriched LDs and is associated by ApoE. Packaging of the capsid takes place with viral budding into the ER lumen at VLDL synthesis sites, mediated by microsomal triglyceride transfer protein (MTP). ApoB is lipidated by the MTP to generate VLDL precursors, which further fuse with ER luminal ApoE-bound LDs to form VLDL [87]. The matured HCV is associated with VLDL, in the form of LVP, and secreted through the VLDL secretion pathway. Graphics in this figure were created with BioRender.com (accessed on 26 February 2022).

The importance of cellular lipid and cholesterol in the HCV life cycle has also been documented. HCV entry into hepatocytes, as well as the replication, assembly, and secretion of HCV, are illustrated in Figure 2. It has been indicated that cellular surface proteins CD81 and scavenger receptor B type I (SR-BI) are required elements for the entry of HCV into host cells [72,73,74]. As mentioned, cholesterol is a major component of lipid rafts. Many cellular proteins, including viral receptors, are preferentially localized in lipid rafts (Figure 2a). A study by Kapadia et al. demonstrated that cellular cholesterol content is crucial for HCV entry, and this might be due to the effects on cell surface expression and localization of CD81 [8]. Localization for both CD81 [75,76] and SR-BI [77] to lipid rafts has been demonstrated. Further, direct physical interaction between CD81 and cholesterol has been observed [78], and HCV pseudo-particle fusion with liposomes, an important step for virus entry, was enhanced with the presence of cholesterol in the target membranes [79]. In addition, the cellular Niemann–Pick C1-like 1 (NPC1L1) cholesterol uptake receptor has been identified as a HCV entry factor [80]. Research shows that NPC1L1 expression is required for HCV infection. Silencing or blocking of NPC1L1 impaired the initiation of HCV infection, and the application of NPC1L1 antagonist ezetimibe strongly blocked HCV uptake in vitro [80]. 

It was suggested that HCV might also be able to enter the host cells via an CD81 independent route [81]. This entry pathway associates with the very-low-density lipoprotein receptor (VLDLR), which is important in cholesterol and apolipoprotein uptake [82]. Lipoproteins, lipids, and cholesterol play important roles in virus production and secretion (Figure 2b). HCV replication complex was found to associate with cholesterol-enriched lipid rafts in the cells [83]. Disruption of the cholesterol synthetic pathway by inhibiting HMG-CoA reductase, the enzyme catalyzing the rate limiting step, completely abolished HCV replication, according to [84]. Research shows that LDs are essential organelles involved in the production of infectious HCV particle assembly [15,85]. Disrupting the HCV core protein–lipid droplet associations leads to a loss in infectious virus production [16]. HCV core protein, responsible for viral capsid formation, is targeted to LDs by its relatively hydrophobic domain 2. A study comparing the HCV core protein sequences of two HCV strains, JFH1 and Jc1, differed in their virus production efficiencies in cultured human hepatoma cells, finding that the amino acid differences in the HCV core protein domain 2 could cause significant differences in the HCV core protein accumulation on LDs, leading to differences in the efficiency of virus production [17]. As the infectivity and productivity of HCV is highly related to lipid and cholesterol metabolism in host cells, these metabolic pathways are considered as drug targets in the treatment of HCV infection [86].

## 4. Therapeutic Potential of Natural Phytochemicals Targeting Cholesterol Metabolism for Treating Viral Infections

**Figure 3:**
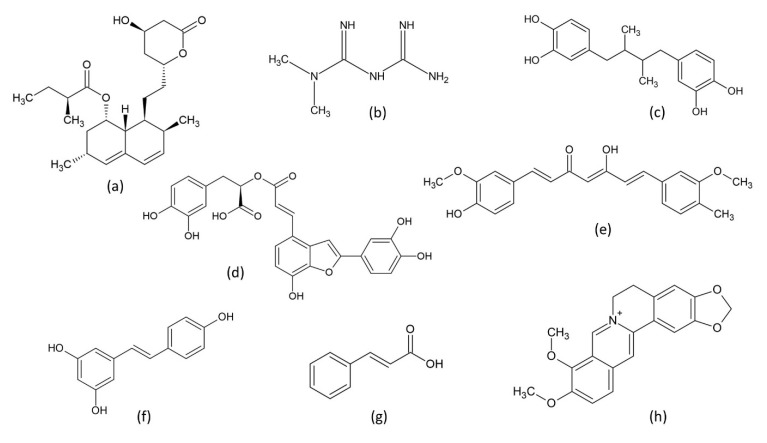
Chemical structures of selected natural compounds with inhibitory activities on cholesterol biosynthesis. (**a**) lovastatin; (**b**) metformin; (**c**) nordihydroguaiaretic acid; (**d**) salvianolic acid C; (**e**) curcumin; (**f**) resveratrol; (**g**) cinnamic acid; (**h**) berberine.

Chronic hepatitis, compared with acute hepatitis, can cause more serious problems, such as liver fibrosis, cirrhosis, and even HCC. As HBV and HCV often cause chronic hepatitis, research scientists have developed (are developing) strategies to deal with their infections. As discussed previously, lipid and cholesterol metabolism and homeostasis are crucial for hepatitis viral infection; cholesterol/lipid metabolism have been identified as potential antiviral targets [90]. Indeed, cellular cholesterol has been identified as a critical factor in a variety of viral infections [91,92,93,94,95,96]. Research shows that several FDA-approved cholesterol-lowering drugs exhibit antiviral abilities against flaviviruses, including dengue, Zika, yellow fever, and West Nile viruses [97]. Statins are fungal origin molecules capable of inhibiting HMG-CoA reductase, thus inhibiting cholesterol synthesis in cells [98,99]. Statins, with their strong cholesterol-lowering abilities, have been applied in the treatment of hypercholesterolemia [98]. There have been several attempts to use statins in the treatment of dengue viral infections. Cell-level experiments have shown that the use of lovastatin (Figure 3a), the first marketed statin drug used to treat high blood cholesterol and reduce the risk of cardiovascular disease, inhibited dengue virus RNA replication and viral secretion [100,101,102,103,104]. In a dengue virus-infected mouse model, lovastatin treatment was found to delay viral infection and increase the survival rates of infected mice [102]. However, a randomized, double-blind, placebo-controlled human clinical trial found that, although lovastatin is safe and well tolerated in adults with dengue viral infection, lovastatin treatment was unable to inhibit dengue viral infections in patients [105].

Metformin (Figure 3b), a natural phytochemical originally found in the plant *Galega officinalis* [106], currently serves as a first-line medication for the treatment of type 2 diabetes [107]. Metformin is known to activate 5′ AMP-activated protein kinase (AMPK), which plays a key role in cellular energy homeostasis. Activation of AMPK results in downregulation of HMG-CoA reductase activity, consequently suppressing cholesterol biosynthesis [108]. The suppression of cholesterol synthesis might be linked to antiviral effects. This argument was supported by a study by Soto-Acosta et al. in which significant inhibition of the dengue virus replication was observed in cells treated with metformin [109]. Similar effects were also observed in experiments using other AMPK activating agents on other flaviviruses. Nordihydroguaiaretic acid (Figure 3c), a lignan found in *Larrea tridentate* with AMPK activation activity [110], and its derivative, were found to be effective against dengue [111], West Nile, and Zika viruses [112]. Another AMPK activator PF-06409577, an indole acid exhibiting cholesterol-lowering activity [113,114], was also found to inhibit flavivirus infection; this inhibition was through modification of host cell lipid metabolism [115]. Collectively, these results indicate that lowering cholesterol levels in the hosts can be a promising strategy for the treatment of flavivirus infections.

As HCV is also a flavivirus, the manipulation of cholesterol synthesis might also be a feasible strategy for treating HCV infection. As mentioned, statins are powerful inhibitors of cholesterol synthesis. The antiviral activity of lovastatin against HCV was reported by Ye et al. [84] and Kapadia and Chisari [116]. Ikeda and co-workers tested anti-HCV profiles of several statins, and found that atorvastatin, fluvastatin, and simvastatin exhibited stronger anti-HCV activity than lovastatin [86]. They also demonstrated that the combination treatment of interferon α and fluvastatin was a more effective treatment, as compared to that of interferon α with ribavirin [86]. Effects of metformin on HCV infection were also investigated. A randomized controlled clinical trial indicated that the combination of metformin with antiviral therapy increased the sustained virological response rate of patients infected with HCV genotype 1 and with insulin resistance [117].

In addition to HCV, statins were shown to be effective in the inhibition of HBV. Inhibition of cholesterol synthesis in HBV-producing cells using lovastatin resulted in impaired secretion of sub-viral particles, according to [118]. The antiviral activity of simvastatin was reported by Bader and Korba [119], and a combination of simvastatin with each of the FDA-approved nucleoside analogue inhibitors, including lamivudine, adefovir, tenofovir, and entecavir, showed synergistic antiviral activity [119]. As chronic HBV infection is highly related to the development of HCC, the inhibition of prolonged HBV infection might reduce the risk of HCC development in patients. A cohort study showed a dose-response relationship between statin use and the risk of HCC in the HBV cohort [120]. NB-598 is a potent inhibitor of squalene epoxidase [121], which is also a key enzyme in cholesterol synthesis. Cell experiments carried out by Bremer et al. demonstrated that infection with HBV from hepatocyte cultures treated with NB-598 showed a more than 60% decrease in infectivity as compared to HBV obtained from untreated cultures [54]. The indirect inhibitor of HMG-CoA reductase, metformin, was also found to inhibit HBV protein production and replication in human hepatoma cells [122].

Based on the mentioned previous studies, it is possible to apply drugs/molecules to alter cholesterol levels for the purposes of inhibition of viral infections. As hypercholesterolemia is a risk factor for cardiovascular diseases, efforts have been made to develop cholesterol-lowering drugs. HMG-CoA reductase, as a key enzyme in cholesterol synthesis, has been focused on in drug development. In silico approaches have been applied in the discovery of natural compounds exhibiting HMG-CoA reductase inhibitor activities [123], and in the investigations, concerning interactions between identified compounds and the enzyme [124,125]. Suganya et al., by using molecular docking and molecular dynamics simulations, identified rutin, a flavonoid found in a variety of plants, including citrus, to be a strong binder and inhibitor of HMG-CoA reductase [126]. Lin and co-workers applied structure-based screening to identify strong HMG-CoA reductase binding compounds from a traditional herbal medicine database [123]. They identified salvianolic acid C (Figure 3d), curcumin (Figure 3e), and docosanol, along with cell experiments, as possible strong inhibitors of HMG-CoA reductase [123]. Indeed, salvianolic acid C was found to impair structural formation of the envelope bound spike protein of SARS-CoV-2, inhibiting viral infections [127]. Curcumin was found to inhibit HCV entry into human hepatocytes by altering membrane fluidity, impairing virus binding and fusion [128]. The inhibitory effects of curcumin on HBV entry into host cells were also documented [129]. In addition, research shows that curcumin is capable of interfering with the infection processes of dengue [130] and Zika viruses [131].

A variety of natural compounds and extracts have been tested for their inhibitory activities on HMG-CoA reductase in animal models [132]. Experiments using LDLR (-/-) mice fed with high-cholesterol diets found that curcumin treatment lowered plasma and hepatic cholesterol comparable to the effects of lovastatin [133]. Resveratrol (Figure 3f), a stilbenoid and a phytoalexin, was found to attenuate the expression of HMG-CoA reductase mRNA in Syrian golden hamsters [134]. A study by Lin and co-workers found that resveratrol treatments were able to assist in the recovery from a fatty liver and protect against HBV-induced HCC in a mouse model [135]. Cinnamic acid (Figure 3g) is a phenolic compound found in plant materials, such as cinnamon oil, Shea nut (*Vitellaria paradoxa*) oil, and balsams. It is the foundation of substances for the biosynthesis of a variety of plant compounds, including flavonoids, lignols, coumarins, aurones, stilbenes, catechin, and phenylpropanoids. In a rat model, dietary cinnamic acid can result in inhibited hepatic HMG-CoA reductase activity, leading to lower hepatic cholesterol content [136]. An investigation by Amano et al. demonstrated that several cinnamic acid derivatives exhibited inhibitory activity on HCV replication [137]. Berberine (Figure 3h) is a quaternary ammonium salt in plants, such as *Berberis*. Inhibitory effects on HMG-CoA reductase by administration of berberines have been observed in rats [138,139] and hamsters [140]. Research shows that berberine could specifically impede HCV attachment and entry steps of a viral infection [141]. Table 2 presents examples of natural cholesterol-lowering compounds tested to exhibit antiviral activities. As combating hypercholesteremia is one of the tasks involved in medical practice/research, it is believed that the development of novel cholesterol-lowering compounds will continue to grow. As downregulation of cholesterol synthesis can be effective, on its own or in combination with other antivirals, to inhibit viral infections, the development of novel cholesterol-lowering compounds will be beneficial in future treatments of viral hepatitis.

As many pure, cholesterol-lowering compounds from natural sources exhibit antiviral activities, it can be implied that cholesterol-lowering foods or dietary patterns might also be beneficial as supplements or aids to prevent or reduce problems caused by viral hepatitis. With this logic, a high-cholesterol diet might further deteriorate the situation. These concepts have been tested in several studies. It was suggested in an animal model study that a high-cholesterol diet could indeed promote steatohepatitis and liver tumorigenesis in HCV core protein expressing transgenic mice [143]. On the other hand, it was found that black raspberry extract showed protective effects against hypercholesterolemia and subsequently reduced hepatitis in rat models fed with high-fat/high-choline diets [144]. A retrospective cohort analysis showed that chronic hepatitis C patients adhering to fish-rich dietary patterns had lower viral loads [145]. Red yeast rice is a Chinese food product in which rice is fermented with a fungus, *Monascus purpureus*. This product is claimed to have the ability to reduce blood cholesterol and triglyceride levels in humans, as it contains a considerable amount of statins [146]. One study indicated that *Monascus* pigment derivatives were able to inhibit HCV replication by interfering with the mevalonate biosynthesis, the reaction catalyzed by HMG-CoA reductase [147]. However, statins are powerful cholesterol-lowering compounds. An overdose of statins might cause adverse effects [146]. A case of acute liver injure induced in a patient taking a red yeast rice supplement for cholesterol-lowering purposes was reported [148]. Thus, caution is still required, even when following effective dietary patterns or taking natural compounds/extracts for specialized health purposes.

## 5. Conclusions

Viral hepatitis is commonly caused by five hepatitis viruses, including HAV, HBV, HCV, HDV, and HEV. Among these five viruses, HBV and HCV have attracted the most attention in biomedical research, as they often cause chronic infections and inflammation, leading to liver fibrosis, cirrhosis, and sometimes HCC. The infections and life cycles of the hepatitis viruses are heavily associated with the metabolism of lipids, particularly cholesterol. Cholesterol levels in the host play a major role in viral infections; research shows that inhibition of cholesterol synthesis could reduce the infections of a variety of human viruses, including HBV and HCV. Therefore, manipulation of cholesterol biosynthesis by cholesterol-lowering drugs are proposed as a strategy to treat hepatitis viral infections. In the investigations, for this purpose, HMG-CoA reductase, the rate-limiting enzyme in cholesterol biosynthesis, is the most studied target. Owing to the great need to solve health problems caused by hypercholesterolemia, extensive efforts have been made into the discovery/development of cholesterol-lowering medications. Plants are natural sources of therapeutic compounds. Natural phytochemicals with desired cholesterol-lowering activities have been identified, and their efficacies at inhibiting viral infections have been tested. It is expected that, with the increasing number of cholesterol-lowering natural compounds being discovered, more choices could be provided for the design/development of antiviral strategies that will treat/prevent viral hepatitis in the future.

## Figures and Tables

**Table 1:** Characteristics of hepatitis viruses.

	HAV	HBV	HCV	HDV	HEV
Family	*Picornaviridae*	*Hepadnaviridae*	*Flaviviridae*	Not applicable	*Hepeviridae*
Genus	*Hepatovirus*	*Orthohepadnavirus*	*Hepacivirus*	*Deltavirus*	*Orthohepevirus*
Genome	Positive-sense single-stranded linear RNA	Double-stranded DNA	Positive-sense single-stranded linear RNA	Negative-sense single-stranded circular RNA	Positive-sense single-stranded linear RNA
Transmission	Fecal–oral	Exposure to infected blood or body fluid/sexual/perinatal	Exposure to infected blood	Exposure to infected blood/body fluids	Fecal–oral/zoonotic/blood transfusion
Clinical outcome of infection	Self-limited	Self-limited and chronic	Self-limited and chronic	Self-limited and chronic	Self-limited

**Table 2:** Examples of natural cholesterol-lowering compounds tested to exhibit antiviral activities.

Compounds	Original Natural Sources	Functions on Cholesterol Metabolism	Viruses Studied to Be Affected
Statins	fungi	HMG-CoA reductase inhibitor	HBV [118,119], HCV [84,86,116], HEV [7], and other flaviviruses [97,100,101,102,104]
Metformin	*Galega officinalis*	AMPK activator	HBV [122], HCV [117], and other flaviviruses [109]
Nordihydroguaiaretic acid	*Larrea tridentate*	AMPK activator	Flaviviruses [111,112]
Resveratrol	Grapes, blueberries, raspberries, mulberries, and peanuts	HMG-CoA reductase mRNA expression inhibitor	HBV [135]
Salvianolic acid C	*Salvia prionitis*, *Origanum vulgare*, and others	HMG-CoA reductase inhibitor	SARS-CoV-2 [127]
Curcumin	*Curcuma longa* species	HMG-CoA reductase inhibitor	HCV [128], HBV [129], dengue virus [130], and Zika virus [131]
Docosanol	Docosanol is a saturated fatty alcohol found in plants	HMG-CoA reductase inhibitor	Lipid-enveloped viruses, including herpes simplex virus [142]
Cinnamic acid	Cinnamon oil, *Vitellaria paradoxa* oil, and balsams from plants	HMG-CoA reductase inhibitor	HCV [137]
Berberine	*Berberis*	HMG-CoA reductase inhibitor	HCV [141]

## Data Availability

This is a review paper and there are no raw data.
